# Updates on Pulmonary Hypertension

**DOI:** 10.2174/0118743064344024250203101417

**Published:** 2025-02-10

**Authors:** Vivek Paudyal, Rubi Thapa, Sagarika Basnet, Munish Sharma, Salim Surani, Joseph Varon

**Affiliations:** 1 Department of General Practice and Emergency Medicine, Karnali Academy of Health Sciences, Jumla, Nepal; 2 Department of Internal Medicine, Kathmandu Medical College and Teaching Hospital, Kathmandu, Nepal; 3 Department of Medicine, Baylor College of Medicine, Texas, TX, United States; 4 Department of Medicine, Texas A & M University, Texas, TX, United States; 5 College of Medicine, University of Houston, Houston, United States

**Keywords:** Pulmonary hypertension, Sotatercept, Inhaled Treprostinil, Tadalafil, Macitentan, Remote pulmonary artery pressure monitoring

## Abstract

Pulmonary Arterial Hypertension (PAH) is an uncommon condition with high mortality. It is an underrecognized condition both in developing and developed countries, especially in developing countries, due to a lack of advanced healthcare facilities and resources for timely diagnosis. More than half of the individuals diagnosed with PAH live less than five years after diagnosis. In recent years, tremendous advancements have been made in diagnostic and therapeutic strategies for PAH patients. Phosphodiesterase 5 (PDE5) inhibitors, endothelin receptor antagonists, and prostacyclin inhibitors in various forms (oral, inhaled, intravenous, or subcutaneous) have been the cornerstone of medical treatment. Atrial septostomy, heart and lung transplant, balloon pulmonary angioplasty, and pulmonary thromboendarterectomy are existing therapeutic options currently available. There has been a continuous effort to introduce newer therapies to improve life expectancy and modify disease. Newer therapies have shown promising results but require future data to guarantee long-term safety and efficacy. We aim to discuss a few of these critical updates in the constantly evolving field of PAH.

## 1 INTRODUCTION

Pulmonary Hypertension (PH) is a spectrum of diseases affecting the pulmonary vessels. Hemodynamically, it is defined as the mean Pulmonary Artery Pressure (mPAP) at rest more significant than 20 mmHg [1, 2].

About 1% of the world’s population is affected by pulmonary hypertension, where 80% of the diseased individuals reside in developing countries. It is most likely underestimated in developing nations as gold standard diagnostic modalities such as right heart catheterization may not be readily available in resource-limited settings. PH, in general, affects the older population, with an average age of 68.5 years and female predominance [3]. Left-sided heart failure is the leading cause of PH worldwide, followed by lung diseases [4]. (Table **1**) depicts the prevalence of different PH groups [3]. The prevalence of PH is increasing and is expected to do so for a few more decades as the aging population is increasing [3, 4].

PAH is a deadly condition, with approximately one-third of the total PAH population dying within the first year of diagnosis and more than half of deaths occurring within five years [3].

Recent advances in drugs and surgical intervention have shown some survival benefits, especially in cases of Group 1 and Group 4 PH [5, 6]. PAH-specific therapies targeting various biological pathways like endothelin, prostacyclin, and phosphodiesterase are currently being used and have improved 5-year survival by up to 60% compared to 34% in 1991 [5]. Still, 40% of patients die within five years of treatment, which highlights the need for the introduction of newer therapies for the management of PAH.

This article discusses the latest updates in managing Pulmonary Arterial Hypertension (PH), as well as Group 2 and Group 3 PH.

### 1.1 Sotatercept: A Novel Decoy Activin Receptor for Pulmonary Arterial Hypertension

PAH is a debilitating, progressive disease characterized by perivascular inflammation and pulmonary vascular remodeling [7].

**Fig. (1):**
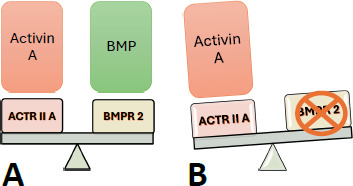
**A**) Figure showing the balance between Activin A and BMP. **B**) The figure shows the pro-proliferative action of Activin A due to a mutation in the BMP gene.

About 43% of PAH cases are either Idiopathic or Heritable, followed by Connective Tissue diseases, which affect about 15% [8]. Mutations affecting Bone Morphogenetic Protein Receptor 2 (BMPR 2), a member of the Transforming Growth Factor β (TGF-β) superfamily, are found in cases of Heritable PAH and sporadic cases [9]. In healthy individuals, BMPR 2 is expressed abundantly in pulmonary vascular endothelium and on the airway and arterial smooth muscles, whereas, in patients with PAH expressing heterozygous mutation of BMPR 2 genes, there is a marked reduction in expression of BMPR 2 [10]. Bone Morphogenetic Protein (BMP) is anti-proliferative and inhibits smooth muscle proliferation and extracellular matrix synthesis, ultimately preventing pulmonary vascular remodeling [11]. It counteracts the pro-proliferative action of Activin A (another member of the TGF-β superfamily), maintaining the balance required for the functioning of the pulmonary vasculature properly [12-14]. Activin A, which functions by binding to Activin Receptor Type II A (ActRIIA), showed enhanced expression in distal pulmonary arterioles in patients with Idiopathic and Heritable PAH [14]. The novel medicine Sotatercept targets the imbalance of Activin A and BMP caused by the BMPR 2 mutation. (Fig. **1**) illustrates the imbalance between these two members of the TGF-β superfamily.

Sotatercept is a fusion protein (ActRIIA-Fc) composed of the extracellular domain of human Activin Receptor Type II A fused with the Fc portion of IgG1. It acts as a decoy receptor for Activin A, inhibiting its binding to ACTR II A and reducing proliferation [14, 15]. It is an FDA-approved drug with two completed clinical trials, i.e., PULSAR Trial (Phase 2, double-blinded, randomized controlled trial followed by open-label extension) and STELLAR Trial (Phase 3, double-blinded, randomized controlled trial) [16].

Both the PULSAR and STELLAR Trials included patients with the diagnosis of PAH, excluding those due to Portal Hypertension, Schistosomiasis, HIV, or veno-occlusive diseases. These candidates were under stable background therapy with PAH drugs for at least three months before the trial and continued their treatment with those drugs throughout the trial [17]. Patients with WHO Functional Class II and III and Pulmonary Vascular Resistance (PVR) ≥ 5 Woods Unit (WU) were only included [15, 17, 18].

In the Phase 2 trial, three groups underwent treatment with 0.3 mg/kg Sotatercept, 0.7 mg/kg Sotatercept, and placebo, whereas in the Phase 3 trial, the initial dose was 0.3 mg/kg, which was later increased to 0.7 mg/kg. Sotatercept was injected every three weeks via a subcutaneous route for 24 weeks, and outcomes were studied [15, 17, 18]. Most of the participants included were white, with more than half suffering from Idiopathic PAH. 37% of participants were using Prostacyclins as stable background therapy. Patients with monotherapy, double treatment, and triple therapy were 9%, 35%, and 56%, respectively [15].

Sotatercept showed significant improvement in pulmonary vascular resistance (PVR) and World Health Organization (WHO) Functional class [15-18]. The least squares mean change from baseline in PVR was a decrease of 2 Wood units (162.2 dynes/sec/cm) in subjects in the sotatercept 0.3 mg group and 3.2 Wood units (255.9 dynes/sec/cm) in the sotatercept 0.7 mg group. In contrast, a decrease of 0.2 Wood units (16.4 dynes/sec/cm) was only observed in the placebo group. These results were consistent across the prespecified subgroups, such as monotherapy vs combination therapy, and whether the patient was receiving Prostacyclin [15]. The median improvement in 6-minute walk distance (6MWD) was 34.4 m in the Sotatercept group compared to the placebo group, which showed an improvement of only 1 m [17]. There are marked improvements in mean Pulmonary Arterial Pressure (mPAP) and NT-pro BNP levels (Table **2**) [16].

The effects observed in the treatment course of 24 weeks with Sotatercept were found to be maintained with continued treatment even for 24 months [18]. About 92% of patients continued the treatment until the end of 24 months, an open-label extension period of the PULSAR Trial [18].

Most participants tolerated the drug. The concerning adverse effects are depicted in Fig. (**2**) [17].

Erythrocytosis and thrombocytopenia were the most concerning adverse events during treatment with Sotatercept, yet these were managed during the trial with dose interruptions or reductions without the need for discontinuation. Epistaxis and gingival bleeding were the most common bleeding events but were mild [17]. Telangiectasia appears to be one of the common side effects, but its clinical significance in long-term management is yet to be determined.

Only 15% suffering from connective tissue diseases were included in the STELLAR trial. PAH associated with Connective Tissue Disease could be of particular concern for treatment with Sotatercept as the risk of bleeding, telangiectasia, and thrombocytopenia is higher in this group. The concomitant use of multiple drugs for the treatment of Connective Tissue Diseases and their interaction is another matter of concern. Similarly, patients with congenital heart disease carry the inherent risk of erythrocytosis, which can be accentuated by Sotatercept raising safety concerns. Further Sotatercept trials should shed more light on these concerns.

## 2 COMBINATION PILL OF MACITENTAN AND TADALAFIL: A PIONEER FIXED DRUG COMBINATION THERAPY FOR PULMONARY ARTERIAL HYPERTENSION

Endothelin and Phosphodiesterase pathways are among the principal pathways involved in the pathophysiology of PAH. The pathophysiological overview is illustrated in Fig. (**3**) [19, 20].

Initial combination therapy with Phosphodiesterase 5 inhibitors (PDE5-is) and Endothelin Receptor Antagonists (ERAs) is recommended for Idiopathic, Hereditary, and drug-induced PAH patients with low to intermediate risk of death. Macitentan, an ERA, and Tadalafil, a PDE-5i, in fixed-dose combination, have shown significant improvement in PVR and 6MWD [21].

Macitentan is a non-selective, oral, potent, dual endothelial receptor antagonist with supercilious efficacy compared to other ERAs due to its better tissue penetration and receptor affinity [22-25]. It has shown significant improvement in morbidity and mortality in patients with PAH [26].

Another drug, Tadalafil, is an orally administered, selective inhibitor of phosphodiesterase type-5 enzyme. It has a faster onset and longer duration of action, higher selectivity for PDE5, and increased absorption, generating potential perk over other PDE5is [27, 28]. Moreover, it has an advantage over other PDE5is due to its anti-inflammatory and antioxidant effects, making it productive even for treating hypobaric hypoxia-induced pulmonary hypertension [29]. Tadalafil is associated with improved 6MWD and eventually enhances the quality of life [30, 31]. Side effects are primarily due to the vasodilatory impacts, such as headache (most common side effect, 33% with Tadalafil 40mg), flushing, dyspepsia, and myalgia [30].

PAH combination therapy has an impact on multiple targeted pathways that are responsible for PAH development [32, 33]. One of the best methods is to use various drugs combined in a fixed ratio into a single dosage, known as fixed drug combination (FDC). It helps increase drug adherence due to a reduction in pill burden and cost, concomitantly allowing better clinical outcomes [32]. To target different pathways, a combination therapy of macitentan and Tadalafil (M/T FDC) was introduced as a pioneer FDC drug in the field of PAH. M/T FDC, a film-coated oral tablet comprising 10 mg Macitentan and 40 mg Tadalafil in a fixed dose combination, is indicated for the chronic treatment of PAH. It reduces morbidity in WHO FC II or III patients with Idiopathic PAH, Hereditary PAH, PAH associated with Connective tissue diseases, and PAH associated with Congenital Heart Diseases [32, 33]. The FDA approval of this FDC drug occurred following the outcomes from the pivotal Phase III “A DUE” study [32]. Macitentan was found to reduce the risk of clinical worsening events and hospitalization, whereas Tadalafil showed improved exercise ability [25, 31, 34].

A DUE study was a prospective, double-blinded, multicenter, randomized, actively controlled, parallel-group, adaptive phase III study designed to compare the efficacy and safety of M/T FDC to macitentan and Tadalafil monotherapies in adults with PAH (WHO FC II or III) followed by an Open-label treatment period with FDC therapy. The participants were either treatment naïve (53%) or were on a stable dose of ERA (17%) or PDE5i (30%) for at least three months before the study [32, 35].

In this pilot study, candidates were categorized into three arms: treatment with macitentan/ tadalafil (M/T FDC), macitentan 10 mg monotherapy, and tadalafil 40 mg monotherapy. These oral tablets were administered for 16 weeks to measure the outcomes. Participants enrolled for M/T FDC, Tadalafil monotherapy, and Macitentan monotherapy were 57%, 24%, and 19%, respectively. Most of the participants engaged were white females suffering from Idiopathic PAH (51%). At enrollment, 51% of patients were WHO FC II, and 49% were WHO FC III [32, 35].

This study met its primary endpoint, demonstrating notable improvement in PVR at 16 weeks. M/T FDC treatment resulted in a statistically significant effect with a 29% reduction in PVR compared to macitentan and a 28% reduction in PVR compared to Tadalafil [35].

The adverse effects were predominantly edema (21%), anemia (19%), and headache (18%). The incidence of withdrawal from the study accounted for 8% due to edema and anemia [35]. Critical adverse effects following the use of M/T FDC are shown in Fig. (**4**) [35].

Furthermore, M/T FDC is contraindicated in pregnancy and in patients who are using organic nitrates and guanylate cyclase stimulators such as riociguat. It should also be used cautiously in patients with hepatic impairment, anemia, and cardiovascular pathology. Due to its hepatic effects in a double-blind and combined double-blind/open-label arms study of M/T FDC, 0.9% and 2.2% of patients discontinued the treatment [35].

The combination of Macitentan and Tadalafil could be a promising drug as it helps bridge the gap between guidelines and everyday clinical practice.

## 3 INHALED TREPROSTINIL IS THE SOLE FDA-APPROVED REMEDY, A POSSIBILITY BESIDES SUPPORTIVE THERAPY FOR PULMONARY HYPERTENSION ASSOCIATED WITH INTERSTITIAL LUNG DISEASE

**Fig. (5):**
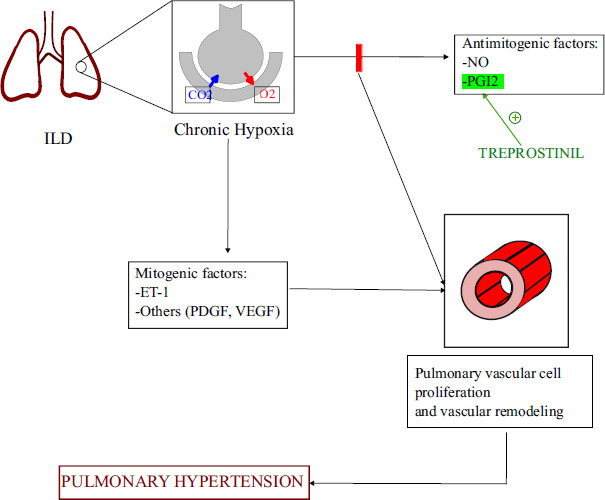
Pathogenesis of ILD complicated by PH along with the mechanism of action of Treprostinil. CO_2_: Carbon dioxide, ET-1: Endothelin 1, NO: Nitric Oxide, O_2_: Oxygen, PDGF: Platelet Derived Growth Factor, PGI_2_: Prostacyclin, VEGF: Vascular Endothelial Growth Factor.

Interstitial lung diseases (ILD) comprise a broad spectrum of diseases usually complicated by pulmonary hypertension during its journey, along with its association with functional impairment, increase in oxygen requirement, decreased quality of life, and poor prognosis. It is included in WHO group 3 pulmonary hypertension [1, 36]. Chronic hypoxia is a crucial factor responsible for the development of PH in patients with respiratory disorders (Fig. **5**) [37-40].

Ever since the launch of inhaled treprostinil, it offered an avenue for the treatment of PH-ILD, as therapeutic choices were often limited to supportive therapy [1]. Treprostinil is a prostacyclin analog [41, 42]. It can be administered orally, intravenously, subcutaneously, or inhalation route [42]. Inhaled Treprostinil demonstrated hemodynamic improvements with reduced PVR and improvement in 6MWD [41]. Furthermore, it is well tolerated due to a few notable adverse effects, such as cough, headache, and sore throat [41, 43, 44]. Adverse effects as per different routes of administration are shown in Fig. (**6**).

Based on the INCREASE trial, inhaled treprostinil is the only FDA-approved treatment considered so far for PH-ILD with survival benefits [1, 40, 45].

The INCREASE trial was a multicenter, randomized, double-blinded, placebo-controlled, phase III trial, accomplished with the prime objective of evaluating the safety and efficacy of inhaled Treprostinil in patients with pre-capillary PH associated with ILD, including combined pulmonary fibrosis and emphysema (CPFE) followed by open-label extension [46]. The primary purpose of the INCREASE trial (Open Label Extension, OLE) was to evaluate the long-term effects of inhaled Treprostinil in PH-ILD [47].

The candidates were categorized equally into two arms: one group received a placebo, and the other received inhaled Treprostinil 12 breaths (6 mcg per breath) four times a day via ultrasonic nebulizer. Outcomes were measured initially at 16 weeks, and the OLE followed for an additional 108 weeks [46, 47]. The maximum dose administered during Open Label Extension was up to 15 breaths per dose of inhaled Treprostinil [47].

Inhaled Treprostinil revealed notable improvements in exercise capacity and decreased clinical worsening (Table **3**) [45, 46]. Prior treatment with inhaled treprostinil was associated with a 31% reduction in the risk of exacerbations as compared to those with prior placebo treatment. It was also found to be safe to use for long-term management [47].

93.3% of the participants from the RCT trial developed adverse effects such as cough (43.6%), headache, dizziness, fatigue, and others, which were quite similar to the previous findings [46]. 94.6% developed adverse effects during the OLE, with 22.3% accounting for drug discontinuation, with higher incidence from candidates who received a placebo in RCT (28.1%) [47]. The safety of the drug during OLE was observed to be consistent with RCT, with improved tolerability over time as evidenced by a reduction in adverse effects like cough and headache experienced by the patients who received inhaled Treprostinil in RCT [47]. Notable adverse effects are demonstrated in Table **4**.

Although this newly approved drug showed significant improvement in patients with PH-ILD with fewer adverse effects, its usual application requires further studies.

## 4 REMOTE PULMONARY ARTERY PRESSURE MONITORING AS A GUIDE FOR THE MANAGEMENT OF HEART FAILURE IN PH PATIENTS

Group 2 PH (PH due to left heart disease) is the most common form of PH with higher mortality as compared to PAH [3]. There is no validated treatment, and its management targets detecting and treating the underlying cause [48].

Pulmonary artery pressure (PAP) monitoring is a hemodynamic-guided heart failure (HF) management system and involves an implantable sensor. It can measure the PAP, and the recordings can be assessed remotely. PAP is a promising early marker for congestion, and its value can guide management plans before clinically evident heart failure ensues [49]. Two types of PAP monitoring devices are currently used: the CardioMEMS HF system (Abbott, Sylmar, CA, USA) and the Cordella PAP Sensor (Endotronix, Inc., Chicago, IL, USA) [49].

The CHAMPION Trial was the first randomized controlled trial assessing the safety and efficacy of the CardioMEMS HF system comprising 550 subjects from 64 different sites in the United States [49, 50]. It included patients diagnosed with Heart Failure for more than three months and falling under New York Heart Association (NYHA) Class III during screening. Eligible candidates were those with hospitalization for decompensated Heart Failure within the past 12 months, irrespective of HF being either systolic or diastolic type. All enrolled candidates received HF sensor implants and were randomized into treatment and control groups before being discharged from the hospital after overnight observation. Treatment groups were provided with standard-of-care HF management as guided by hemodynamic information. In contrast, the control group received standard-of-care HF management, as directed by clinical findings, without using hemodynamic information [50].

Hospitalization for heart failure within six months was reduced by 28% in the treatment group. There was a reduction in 1 hospitalization for every eight patients being treated. The effect was more pronounced during a mean follow-up of 15 months, where the reduction in hospitalization was 37%, requiring only four patients to be treated to decrease one hospitalization [51].

After the randomized access period was completed, all service providers received pulmonary artery pressure information for an additional 13 months (open access period). Patients in the former control group experienced a 48% reduction in hospitalization for heart failure during the open access period compared to hospitalizations of the control group during the randomized period [52]. There were 8 (1%) Device or System-related complications with no sensor failures, suggesting the device's safety profile [51].

Patients with Pulmonary Hypertension have a higher hospitalization risk than HF cases without PH. The management of HF following ambulatory hemodynamic monitoring has shown a significant reduction in Hospitalizations for PH Patients (HR 0.64, 95% CI 0.51 to 0.81, p = 0.002) [53].

Concurrent Pulmonary Artery Pressure monitoring, along with the assessment of clinical signs and symptoms, enabled improved management of heart failure and a possible reduction in hospitalizations. Remote PAP monitoring is a boon for PH patients as it helps in the early detection of raised PAP and helps in managing it before PH progresses.

The CHAMPION trial's limitation was maintaining patient masking and minimizing the effect of investigator-patient and device-patient interactions on outcome. It was not powered to detect a mortality benefit, and more extensive trials of hemodynamic monitoring are needed.

## CONCLUSION

Patients with PAH have high morbidity and mortality despite the optimization of the currently available treatment strategies. New therapeutic and monitoring approaches are necessary to improve patient outcomes.

Sotatercept is a promising add-on therapy for patients with WHO FC II-III symptoms on stable background therapy. Still, long-term data are needed to establish its safety, efficacy, and role in modifying disease (decreasing mortality). Cost affordability and medical insurance coverage could be a few other issues in developing nations, while the availability of these medications could be a challenge in resource-limited countries.

A fixed-dose combination of macitentan and tadalafil has shown supremacy in reducing PVR, but its hepatic side effects are still a concern.

Inhaled Treprostinil in PH-ILD helps the 6MWD. Its route is preferable over other routes of administration.

Remote pulmonary artery pressure monitoring provides valuable information for monitoring HF and PH therapy. More extensive trials are needed before its routine application in mainstream care.

Future data can only tell us if these newer therapies will deliver promising results and help in improving patient survival and quality of life.

## Figures and Tables

**Fig. (2):**
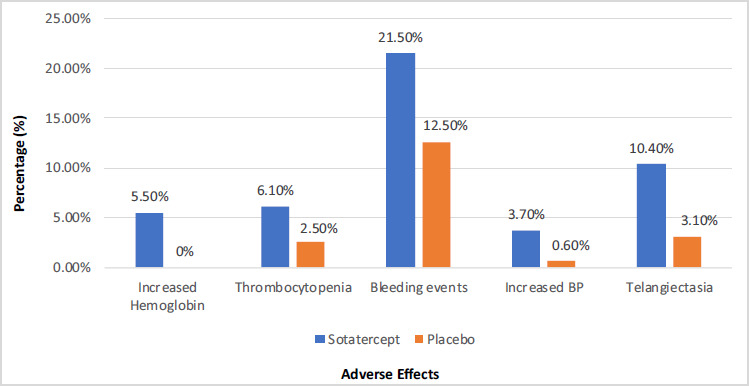
Adverse events occurring at a higher frequency in the Sotatercept group.

**Fig. (3):**
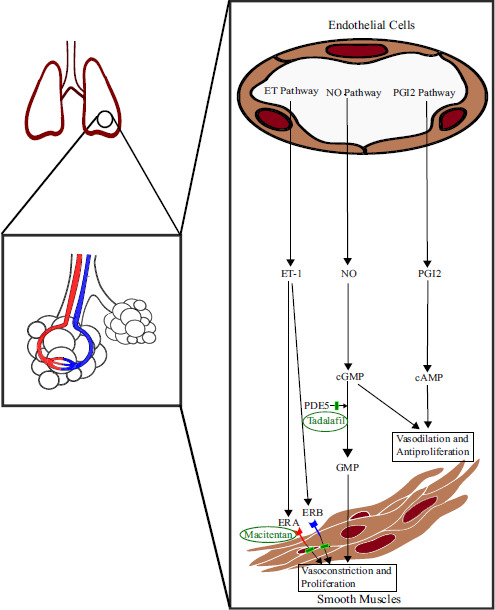
Pathophysiology of PAH, including the targeted site of action of Macitentan and Tadalafil [1, 3]. cAMP: cyclic Adenosine Monophosphate, cGMP: cyclic Guanosine Monophosphate, ET_A_: Endothelin A receptor, ET_B_: Endothelin B receptor, NO: Nitric Oxide, PDE5: Phosphodiesterase 5, PGI_2_: Prostacyclin.

**Fig. (4):**
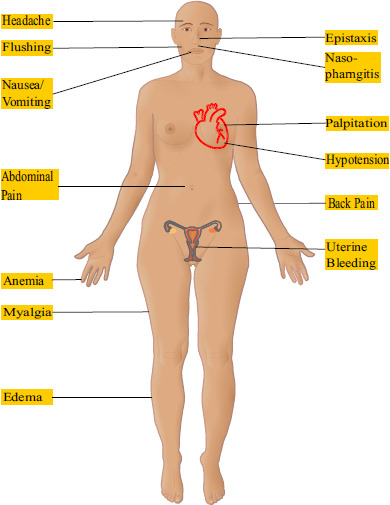
Adverse effects in patients treated with M/T FDC during the 16-week double-blind study portion of A DUE study [35]; modifications in Images by Goran tek-en (CC-BY-SA 4.0), Mikael Haggstrom (CC0).

**Fig. (6):**
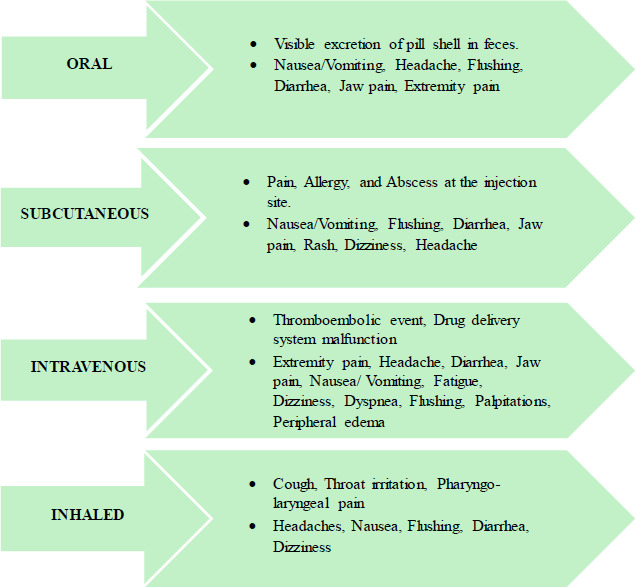
Adverse effects following administration of Treprostinil via different routes [44].

**Table 1:** WHO clinical classification of pulmonary hypertension and its prevalence [3].

**WHO Clinical Groups**	**Prevalence (%)**
**Group 1: Pulmonary Arterial Hypertension (PAH)**	13.8
**Group 2: PH due to left heart disease**	68.5
**Group 3: PH due to lung diseases and Hypoxia**	47
**Group 4: PH due to pulmonary artery obstructions**	9

**Table 2:** A pooled analysis of PULSAR and STELLAR Trials showing effective outcomes of different parameters [16].

**Parameters**	**Mean Deviation (MD)**	**95% Confidence Interval (CI)**
PVR improvement	-229.77	-319.39; -140.14
Right Atrial Pressure	-2.29	-2.77; -1.81
Mean Pulmonary Arterial Pressure (mPAP)	-11.85	-15.31; -8.37
NT-pro BNP levels	-1368.83	-2512.28; -225.39
WHO FC performance improvement	RR: 2.06	1.39; 3.04

**Table 3:** Change from baseline 6MWD and occurrence of clinical worsening from INCREASE RCT trial over 16 weeks [46].

	**Inhaled Treprostinil (N=163)**	**Placebo (N=163)**	**Treatment Effect (95% CI)**	**p-value**
**Change in 6MWD from baseline.**	21.8±5.12	-10.04±5.12	31.12±7.25 (16.85 to 45.39)	<0.001
**Occurrence of clinical worsening (%)**	22.7	33.1		

**Note: **6MWD: 6 Minute Walk Distance, CI: Confidence Interval.

**Table 4:** Adverse effects due to inhaled Treprostinil for 16 weeks of the study of the INCREASE RCT trial and OLE [46, 47].

**Adverse Effects**	**RCT**		**OLE**	
	Inhaled Treprostinil (N=163) %	Placebo (N=163) %	Inhaled Treprostinil in RCT (N=119) %	Placebo in RCT (N=121) %
**Cough**	43.6	33.1	18.5	35.5
**Headache**	27.6	19.6	10.1	27.3
**Dyspnea**	25.2	31.3	25.2	27.3
**Dizziness**	18.4	14.1	15.1	14.9
**Nausea**	15.3	16.0	15.1	11.6
**Fatigue**	14.1	14.1	15.1	11.6
**Diarrhea**	13.5	11.7	16.8	14.0
**Throat irritation**	12.3	3.7		
**Oropharyngeal pain**	11.0	2.5		

**Note: **OLE: Open Label Extension, RCT: Randomized Controlled Trial.

## Data Availability

Not applicable.

## LIST OF ABBREVIATIONS

BMP: = Bone Morphogenetic Protein
TGF-β: = Transforming Growth Factor β
PH: = Pulmonary Hypertension
PDE5: = Phosphodiesterase 5
PAH: = Pulmonary arterial hypertension
CO_2_: = Carbon dioxide
ET-1: = Endothelin 1
NO: = Nitric Oxide
O_2_: = Oxygen
PDGF: = Platelet Derived Growth Factor
PGI2: = Platelet Derived Growth Factor
PGI_2_: = Prostacyclin
VEGF: = Vascular Endothelial Growth Factor
